# Tempol treatment shows phenotype improvement in *mdx* mice

**DOI:** 10.1371/journal.pone.0215590

**Published:** 2019-04-22

**Authors:** Túlio de Almeida Hermes, Rafael Dias Mâncio, Aline Barbosa Macedo, Daniela Sayuri Mizobuti, Guilherme Luiz da Rocha, Valéria Helena Alves Cagnon, Elaine Minatel

**Affiliations:** Department of Structural and Functional Biology, Institute of Biology, State University of Campinas (UNICAMP), Campinas, São Paulo, Brazil; University of Minnesota Twin Cities, UNITED STATES

## Abstract

Considering potential Tempol effects on *mdx* muscle fibers, in this study we evaluated its effects on relevant dystrophic phenotypic characteristics, such as muscle degeneration, inflammatory process and angiogenesis, which as yet have not been investigated. *Mdx* mice were randomly assigned into three groups: *mdxS*, the control group receiving intraperitoneal (i.p.) injections of saline solution (100μL); *mdxP*, positive control group receiving prednisolone (1mg/kg) by oral gavage; and *mdx*T, treated group receiving i.p. injections of tempol (100 mg/kg). C57BL/10 mice were also used as controls. Tempol treatment promoted gain in muscle strength and reduced myonecrosis and inflammatory response in the dystrophic diaphragm (DIA) and biceps brachii (BB) muscles. No evidence of Tempol's beneficial performance on angiogenesis in DIA and BB *mdx* muscles was found. The findings presented here show that Tempol treatment improves dystrophic phenotype, supporting its use as a potential therapeutic strategy in DMD.

## Introduction

Duchenne muscular dystrophy (DMD) is the most common muscular dystrophy, whose genetic defect is identified in the X chromosome gene that encodes the intracellular protein dystrophin [1]. DMD patients usually show motor difficulties by the age of six and the muscle weakness progresses, leaving patients wheelchair-bound by their teens, with death occurring in their twenties owing to respiratory and cardiovascular failure [1].

The DMD physiopathogenesis involves some mechanisms, such as increased intracellular calcium, exacerbated inflammatory process, oxidative stress and altered angiogenesis. In dystrophic patients and in their experimental model, the *mdx* mice, the high intracellular calcium levels are directly related to the increase of oxidative stress and exacerbated inflammation [2,3]. Together, these factors lead to progressive muscle degeneration observed in dystrophic skeletal muscles. In addition, reduced vascular densities and impaired angiogenesis in the dystrophic muscles were also reported [4,5], which may compromise the regenerative muscular process.

Although several pharmacological treatments have been investigated in an attempt to improve the dystrophic phenotype, the corticosteroids are still the standard treatment prescribed for patients with DMD, but their benefits are modest and have numerous side effects [6]. So, DMD remains without adequate treatment.

Recently, Burns and collaborators (2017) reported that Tempol (4-hydroxy-2,2,6,6-tetramethylpiperidine-1-oxyl) supplementation restores diaphragm force and metabolic enzyme activities in *mdx* mice [7]. However, other relevant DMD phenotypic characteristics, such as muscle degeneration, inflammatory process and angiogenesis, were not investigated after treatment with Tempol. Thus, in this study, we verified Tempol therapy effects on these physiological pathways that can contribute to muscle injury in the dystrophic muscle of *mdx* mice. In addition, considering that the dystrophic muscles show different degrees of the dystrophic phenotype, with the respiratory muscle being more severely affected than the limb muscles [8], all parameters analyzed herein were performed on the diaphragm and biceps brachii muscles.

## Methods

### Animals

Animal housing, handling and all experiments were conducted in compliance with the guidelines of the Brazilian College for Animal Experimentation (COBEA). The protocol was approved by the Ethics Committee on the Use of Animals (CEUA) of State University of Campinas (UNICAMP) (Protocol Number 3937–1).

C57BL/10 (C57BL/10ScCr/PasUnib) and *mdx* (C57BL/10-Dmdmdx/PasUnib) mice were kept under standard conditions of temperature (25°C ± 0.5) and relative humidity (55 ± 1) with 12-h light/ dark cycles, and were allowed free access to standard forage and drinking water ad libitum after weaning.

### Experimental design

M*dx* mice (14 days old) were randomly assigned into three groups: *mdxS*, used as a control, receiving intraperitoneal (i.p.) injections of saline solution (100μL); *mdxP*, used as a positive control, receiving prednisolone (1mg/kg) [9] by oral gavage dissolved in saline solution; and *mdx*T, used as the treated group, receiving i.p. injections of Tempol (100 mg/kg) [10] dissolved in saline solution. All three treatments lasted 14 days and all animals were weighed daily till the end of the treatment in order to adjust the drug dose. C57BL/10 mice (Ctrl group) were used as normal controls and received no treatment. After the treatment, all animals were anaesthetized using a mixture of ketamine hydrochloride (130mg/kg; Francotar, Virbac, Fort Worth, TX, USA) and xylazine hydrochloride (6.8mg/kg, 2% Virbaxil; Virbac), and blood samples and the diaphragm (DIA) and biceps brachii (BB) muscles were collected.

### Grip strength evaluation (n = 10 for each group analyzed; the same animals were used for the histopathological analysis)

Forelimb muscle strength was evaluated with a grip strength meter (New Primer, Sao Paulo, Brazil), as previously described [11,12]. In brief, five measurements were obtained for each animal at the beginning (14 days old) and end (28 days old). Absolute strength was normalized to body weight at 14 and 28 days. After the last measurement, the animals were immediately anesthetized and the DIA and BB muscles were collected for analyses.

### Blood samples (serum; n = 5 for each group analyzed)

#### IL-1β and IL-6 analysis

The samples were quantified using a Quantikine IL-1β and IL-6 ELISA kit (Sigma-Aldrich, St. Louis, MO, USA) according to the manufacturer’s instructions.

#### Alanine aminotransferase (ALT) and aspartate aminotransferase (AST)

The ALT and AST assays were carried out using a commercially available kit (BioClin, Ireland) according to the manufacturer’s instructions. Values are reported as international units per litre.

#### Liver morphology analysis (n = 5 for each group analyzed)

The livers of all experimental groups were analysis using the softwares NIS-Elements/Image and Image Pro-Plus in digital microscopic images obtained with the Nikon Eclipse E-400 microscope (Nikon, Tokyo, Japan) as previously described [13]. In brief, the liver fragments were fixed in Bouin solution for 24h and after that, the samples were embedded in paraffin and the blocks were sectioned at 4 μm and stained with hematoxylin and eosin. The following parameters were observed: mononucleate hepatocyte, binucleate hepatocyte, total hepatocyte cytoplasm, lipid hepatic content, blood vessel and inflammatory process.

### Histopathological analysis (n = 5 for each group analyzed)

#### Anti-mouse IgG-FITC antibody

The cryosections of DIA and BB muscles were incubated with fluorescently labelled immunoglobulin G (IgG) for morphological visualization and quantification of muscle fiber damage. In brief, muscle cryosections (8 μm thick) were preincubated for 30min with 5% bovine serum albumin (BSA) in phosphate-buffered saline (PBS), followed by a 1h incubation with IgG fluorescein isothiocyanate conjugate antibody (anti-mouse; Sigma-Aldrich, St Louis, MO, USA). The number of IgG-positive muscle fibers was expressed as a percentage of the total number of muscles fibers counted in each section (4–5 sections per muscle) from all experimental groups.

#### Hematoxylin & eosin

The cryosections of DIA and BB muscles stained with HE were observed under a light microscope. The evaluated parameters were: number of regenerated fibers, characterized by the centralized nucleus [14], and areas of inflammation (Infl), presenting fibers in the early stage of regeneration, characterized by small basophilic myocytes with central nucleus, interspersed by inflammatory infiltrate, in areas of great cellularity.

The sections were analyzed by light microscopy (Nikon) connected to a Nikon camcorder in 20X objective, through NIS-elements software AR Advances Researches. All the fibers of the cuts (normal and regenerated fibers) were counted to estimate the total population of fibers of each muscle. This allowed the percentage of normal and regenerated fibers of the studied animals to be obtained.

Areas of inflammation (Infl) and total muscle area were also delimited. To delimit these areas, the images were captured through a Nikon® Digital Sight, attached to a light microscope (Nikon®), connected to a computer with NIS-elements software AR Advances Researches.

#### F4/80 staining—Macrophage infiltration

For macrophage infiltration, muscle sections for F4/80 staining were fixed in acetone for 10min, air dried for 20min and washed with PBS. Sections were washed with PBS and blocked for 1h at room temperature with 3% BSA in PBS. The slides were incubated overnight at 4°C with primary antibody against F4/80 (monoclonal antibody; AbD Serotec, Raleigh, NC, USA). After PBS washes, the slides were incubated with anti-rat secondary antibody (Texas Red® Anti-rat IgG; Vector Laboratories, Burlingame, CA, USA) for 1h at room temperature. After washing with PBS, the muscle sections were mounted in 1,4-diazabicyclo[2.2.2]octane (DABCO; Sigma) mounting medium for fluorescence microscopy (Nikon Eclipse TS100). F4/80 staining was quantified using NIS-elements AR Advances Research software. For F4/80 immunofluorescence the percentage of total muscle area was calculated in each section studied (four or five sections per muscle). A blinded observer carried out the counts and measurements.

#### Microvessels density analysis

Microvessel density in DIA and BB muscles cross-sections from C57Bl/10 mice and dystrophic *mdx* mice was determined by counting blood vessels with positive staining for CD31 (rabbit polyclonal IgG; sc1506-r, Santa Cruz Biotechnology), the pattern protocols were in agreement with to those reported by Verma [15]. Briefly, ten random fields without overlap were analyzed per animal with the 40× objective. A Nikon Eclipse E-400 light microscope (Nikon, Tokyo, Japan) was used to photograph these fields which were then submitted to CD31 positive vessel counting by means of Nis-Elements software: Advanced Research (USA). An average value of 10 fields from each animal expressed the microvessel density.

### Western blot analysis (n = 5 for each group analyzed)

The TNF-α and VEGF content in the DIA and BB muscles of all experimental groups was analyzed using Western blotting. An assay lysis buffer containing freshly added protease and phosphatase inhibitors (1% Triton, 10 mM sodium pyrophosphate, 100mM NaF, 10μg/ml aprotinin, 1mM phenylmethanesulphonyl fluoride and 0.25mM Na_3_VO_4_) was used to lyse the muscles. The samples were centrifuged at 11.000 rpm for 20min, and the soluble fraction was resuspended in 50μl Laemmli loading buffer (2% sodium dodecyl sulphate [SDS], 20% glycerol, 0.04mg/ml bromophenol blue, 0.12M Tris-HCl, [pH 6.8] and 0.28M β-mercaptoethanol). A total of 30μg total protein homogenate from each sample was placed onto 12–15% SDS-polyacrylamide gels. Proteins were transferred from the gels to a nitrocellulose membrane using a submersion electrotransfer apparatus (Bio-Rad Laboratories, Hercules, CA, USA). Membranes were blocked for 2h at room temperature with 5% skim milk/Tris-HCl buffer saline-Tween buffer (TBST; 10mM Tris-HCl [pH 8], 150mM NaCl and 0.05% Tween 20). The membranes were incubated with the primary antibodies overnight at 4°C, washed in TBST, incubated with the peroxidase-conjugated secondary antibodies for 2h at room temperature and developed using the SuperSignal West Pico Chemiluminescent Substrate kit (Pierce Biotechnology, Rockford, IL, USA). To control for protein loading, Western blot transfer and nonspecific changes in protein levels, the blots were stripped and re-probed for glyceraldehyde-3-phosphate dehydrogenase (GAPDH). Band intensities were quantified using the GeneTools software (SynGene–A Divison of Synoptics, Cambridge, England).

The following primary antibodies were used for Western blotting: TNF-α (rabbit anti-mouse polyclonal antibody; Millipore, CA, USA); VEGF (goat polyclonal antibody; Santa Cruz Biotechnology); and GAPDH (rabbit polyclonal antibody; Santa Cruz Biotechnology). The secondary antibody was peroxidase-labelled affinity-purified mouse or rabbit IgG antibody (KPL, Gaithersburg, MD, USA).

### Statistical analysis

All data are expressed as mean ± standard deviation (SD). Statistical analysis for direct comparison between means of groups was performed by ANOVA, followed by Tukey test used for multiple statistical comparisons between groups. *p*≤0.05 was considered statistically significant.

## Results

### Tempol toxicity analyses

In order to analyze the possible Tempol toxicity on C57BL/10 and *mdx* mice, we evaluated, throughout the treatment, the body weight, liver morphology and changes in liver function by alanine aminotransferase (ALT) and aspartate aminotransferase (AST) levels.

**Fig 1:**
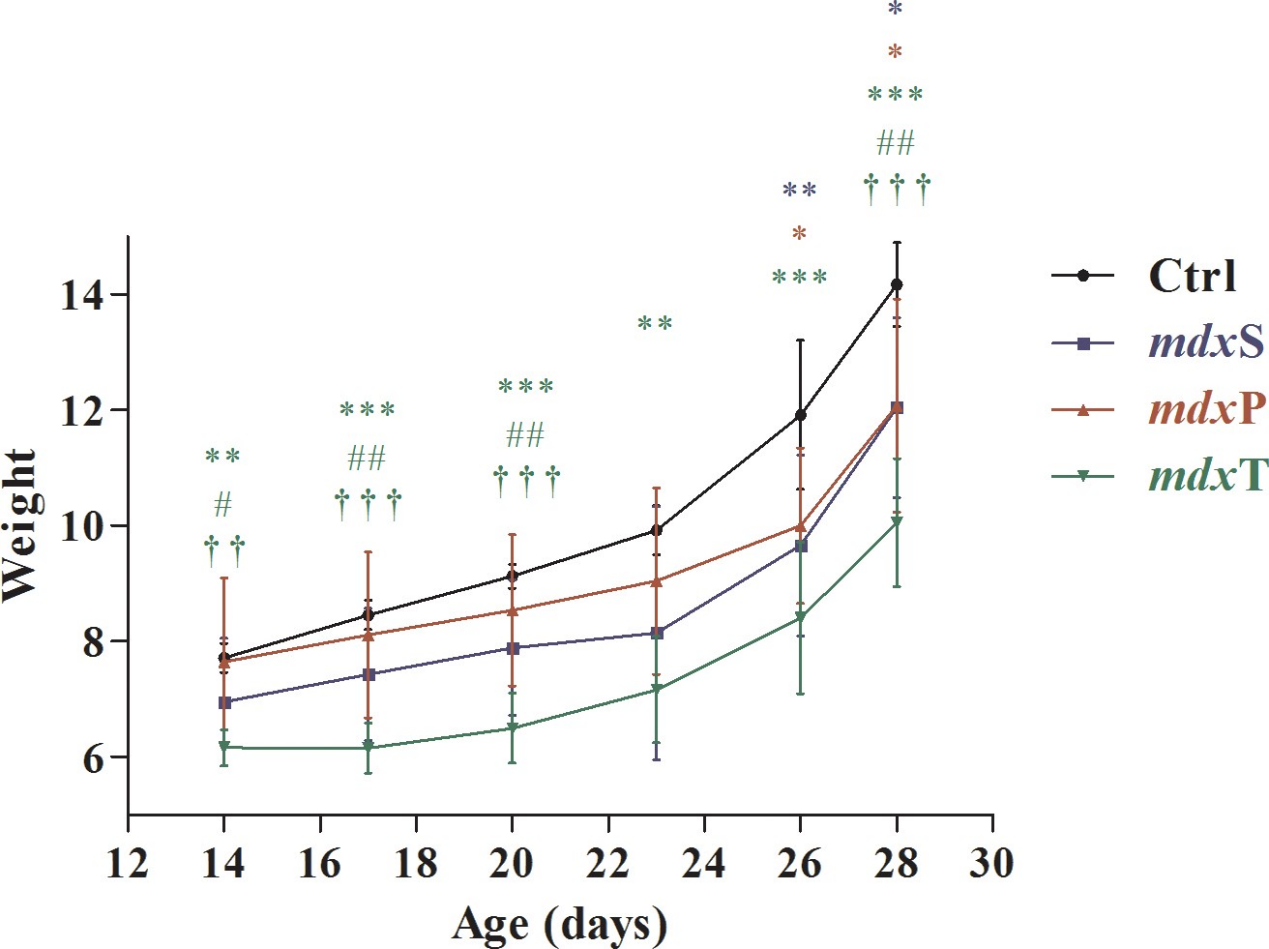
Weight (g) in control and dystrophic mice during the treatment period. C57BL/10 mice (Ctrl; black line), saline-treated *mdx* mice (*mdx*S; blue line), prednisolone-treated *mdx* mice (*mdx*P; red line) and Tempol-treated *mdx* mice (*mdx*T; green line). All values expressed as mean ± standard deviation (SD). *P ≤ 0.05 compared with Ctrl group, **P ≤ 0.001 compared with Ctrl group, ***P ≤ 0.0001 compared with Ctrl group, ^#^P ≤ 0.05 compared with *mdx*S group, ^##^P ≤ 0.001 compared with *mdx*S group, ††P ≤ 0.001 compared with *mdx*P group, †††P ≤ 0.0001 compared with *mdx*P group (one-way ANOVA with Tukey’s post-hoc test).

All experimental groups presented weight gain during the experimental period (by 83.7% in the ctrl mice group; 73.3% in the *mdxS* mice group; 58.1% in the *mdxP* mice group and 63.1% in the *mdxT* mice group; Table 1). The treatment with prednisone presented the lower weight gain in the period, which was significantly different from the control and Tempol-treated *mdx* groups (Fig 1). The Tempol-treated *mdx* mice also presented statistical difference from the control saline *mdx* mice, but these animals already had lower weight at the beginning of the treatment (Fig 1).

**Table 1:** Body weights and forelimb muscle strength.

	Body weight (g)		Body weight/period	Force/Body weight (g/g)		Force gain/period
	Start	End	(%)	Start	End	(%)
**Ctrl**	7.7±0.2	14.2±0.7	83.7	2.8±0.4	3.3±0.6	18.4
***mdx*S**	7.0±1.1	12.0±1.6**	73.3	2.0±0.4**	2.2±0.4***	11.3
***mdx*P**	7.6±1.4	12.1±1.8***	58.0	2.4±0.3	2.9±0.3^#^	23.6
***mdx*T**	6.2±0.3**^,^^#^^,^††	10.1±1.1***^,^^###^	63.1	2.0±0.2***	2.8±0.4^##^	44.5

Body weight (g) was measured at the beginning (Start) and after 2 weeks (End) of Tempol treatment. Body weight/period: Percentage the weight gain on the treatment period (%). Forelimb muscle strength was assessed by taking measurements of force at Start and End, normalized by body weight (g/g). Gain Force/period: Percentage the muscular force gain on the treatment period (%). Experimental groups: C57BL/10 mice (Ctrl), saline-treated *mdx* mice (*mdx*S), prednisolone-treated *mdx* mice (*mdx*P) and Tempol-treated *mdx* mice (*mdx*T).

All values are shown as mean ± standard deviation (SD) for 10 animals (n = 10).

**P ≤ 0.001 compared with Ctrl group

***P ≤ 0.0001 compared with Ctrl group

^#^P ≤ 0.05 compared with *mdx*S group

^##^P ≤ 0.001 compared with *mdx*S group

^###^P ≤ 0.0001 compared with *mdx*S group

††P ≤ 0.001 compared with *mdx*P group (one-way ANOVA with Tukey’s post-hoc test).

**Fig 2:**
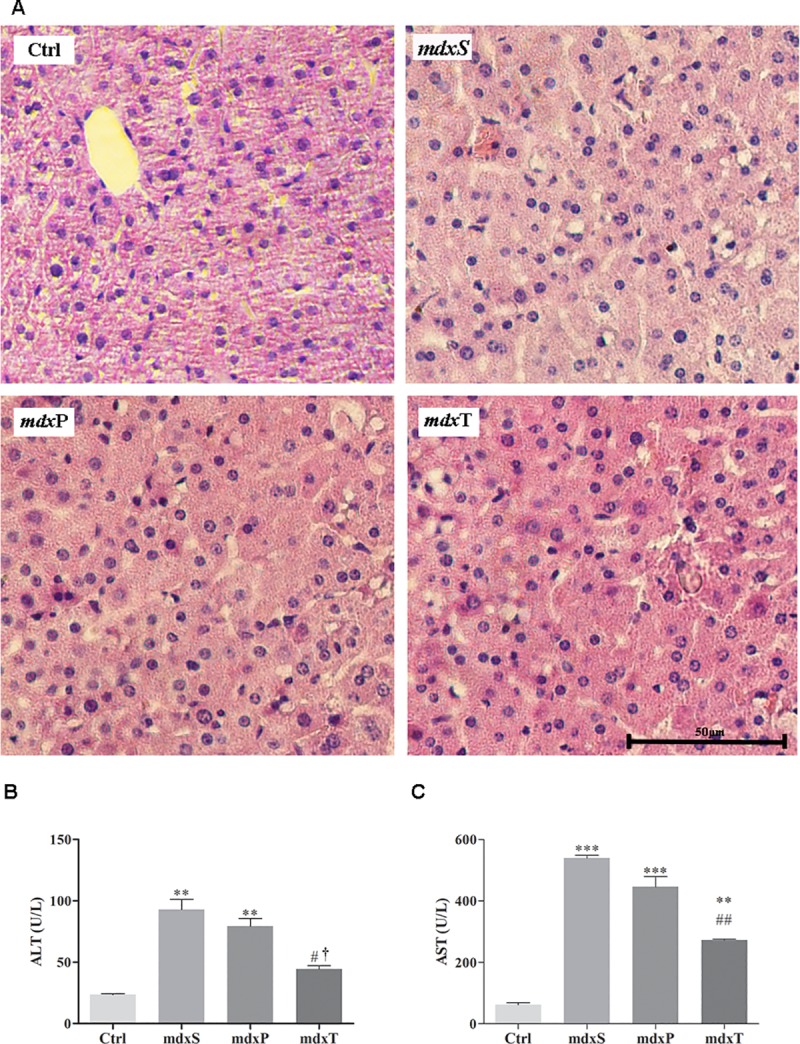
Liver analysis in control and dystrophic mice. In (A) photomicrographs of liver stained with hematoxylin and eosin in C57BL/10 mice (Ctrl), saline-treated *mdx* mice (*mdx*S), prednisolone-treated *mdx* mice (*mdx*P) and Tempol-treated *mdx* mice (*mdx*T). Bar = 50 μm. In (B) detection of ALT levels and in (C) detection of AST levels in serum of Ctrl, *mdx*S, *mdx*P and *mdx*T. **P ≤ 0.001 compared with Ctrl group, ***P ≤ 0.0001 compared with Ctrl group, ^#^P ≤ 0.05 compared with *mdx*S group, ^##^P ≤ 0.001 compared with *mdx*S group, †P ≤ 0.05 compared with *mdx*P group (one-way ANOVA with Tukey’s post-hoc test).

Liver morphology did not differ significantly between all experimental groups. As shown in Fig 2, all groups showed normal liver morphology, characterized by hepatocytes, displayed a polygonal format with mononuclear or binuclear central nuclei, arranged in anatomizing plates, with borders that faced either sinusoid capillaries or adjacent hepatocytes. Any inflammatory infiltrates were observed in the hepatic parenchyma.

Although ALT and AST levels in *mdx* mice (ALT: 92.9±12U/L and AST: 359.5±12.3U/L) were higher than the control group (ALT ctrl: 23.6±1.2U/L; AST ctrl: 61.9±8.6U/L), Prednisolone (ALT: 79.4±8.7U/L; AST: 445.7±47.7U/L) and Tempol (ALT: 44.5±3.7U/L; AST: 272.3±4.9U/L) treatments significantly decreased these levels (Fig 2).

### Tempol effects on muscle strength

Regarding muscle strength over the study period, the *mdx*S group showed the lowest gain and statistically differed from the control group. The *mdx*T group showed the best strength gain (by 44.5%) at the end of the study (Table 1).

### Tempol effects on degeneration/regeneration process

**Fig 3:**
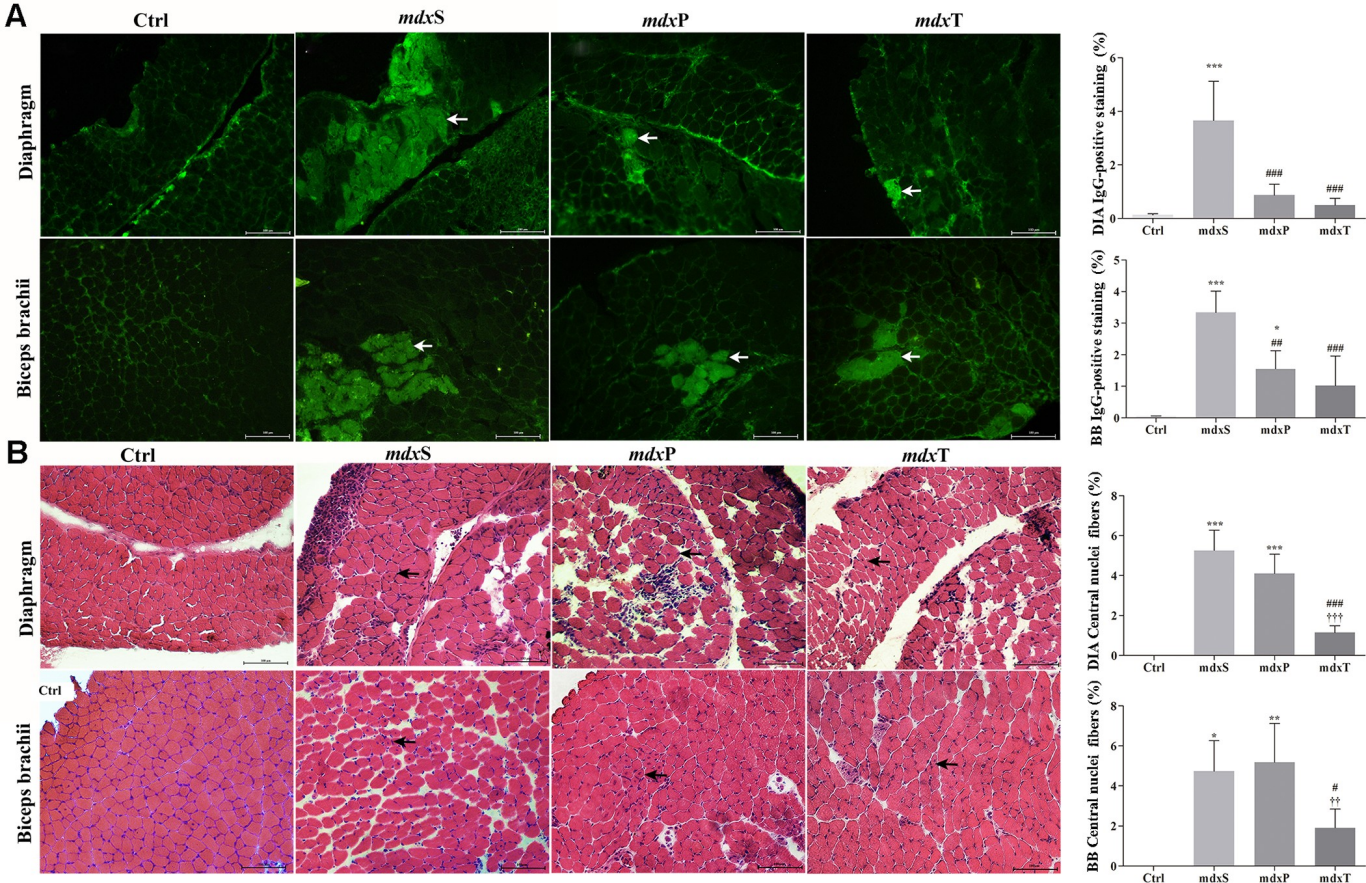
Degeneration/regeneration process in dystrophic diaphragm and biceps brachii muscles. In **A**, diaphragm (DIA) and biceps brachii (BB) cross-sections showing IgG staining (white arrow) in C57BL/10 mice (Ctrl), saline-treated *mdx* mice (*mdx*S), prednisolone-treated *mdx* mice (*mdx*P) and Tempol-treated *mdx* mice (*mdx*T). The graphs show the IgG staining (%) in the DIA and BB muscles of Ctrl, *mdx*S, *mdx*P and *mdx*T. In **B**, DIA and BB cross-sections showing fibers which central nuclei (black arrow) in the DIA and BB muscles of Ctrl, *mdx*S, *mdx*P and *mdx*T. The graphs show the nuclei central fibers in the DIA and BB muscles of Ctrl, *mdx*S, *mdx*P and *mdx*T. All values expressed as mean ± standard deviation (SD). *P ≤ 0.05 compared with Ctrl group, **P ≤ 0.001 compared with Ctrl group, ***P ≤ 0.0001 compared with Ctrl group, ^#^P ≤ 0.05 compared with *mdx*S group, ^##^P ≤ 0.001 compared with *mdx*S group, ^###^P ≤ 0.0001 compared with *mdx*S group, ††P ≤ 0.001 compared with *mdx*P group, †††P ≤ 0.0001 compared with *mdx*P group (one-way ANOVA with Tukey’s post-hoc test).

Sarcolemmal integrity loss was evaluated in DIA and BB muscles of experimental groups by intracellular fiber staining for IgG antibody. Control (C57BL/10) mice showed no intracellular fiber staining (Fig 3A), while *mdx* mice showed intracellular staining for IgG antibody within some muscle fibers (Fig 3A). The *mdx*P and *mdx*T groups showed a significant decrease in IgG staining in the DIA (by 76.9% and 86.8%, respectively) and BB (by 53.5% and 69.4%, respectively) muscles compared to *mdx*S group (Fig 3A).

Regenerated muscle fibers indicated by central nuclei were seen in the dystrophic DIA and BB muscle (Fig 3B). The *mdx*S group demonstrated an increase in the number of regenerated muscle fibers in DIA and BB muscles and the Tempol treatment significantly reduced this increase (Fig 3B). The Prednisolone treatment had no effect on the number of regenerated muscle fibers in the dystrophic muscles compared to muscles from *mdx*S group (Fig 3B).

### Anti-inflammatory Tempol effects

**Fig 4:**
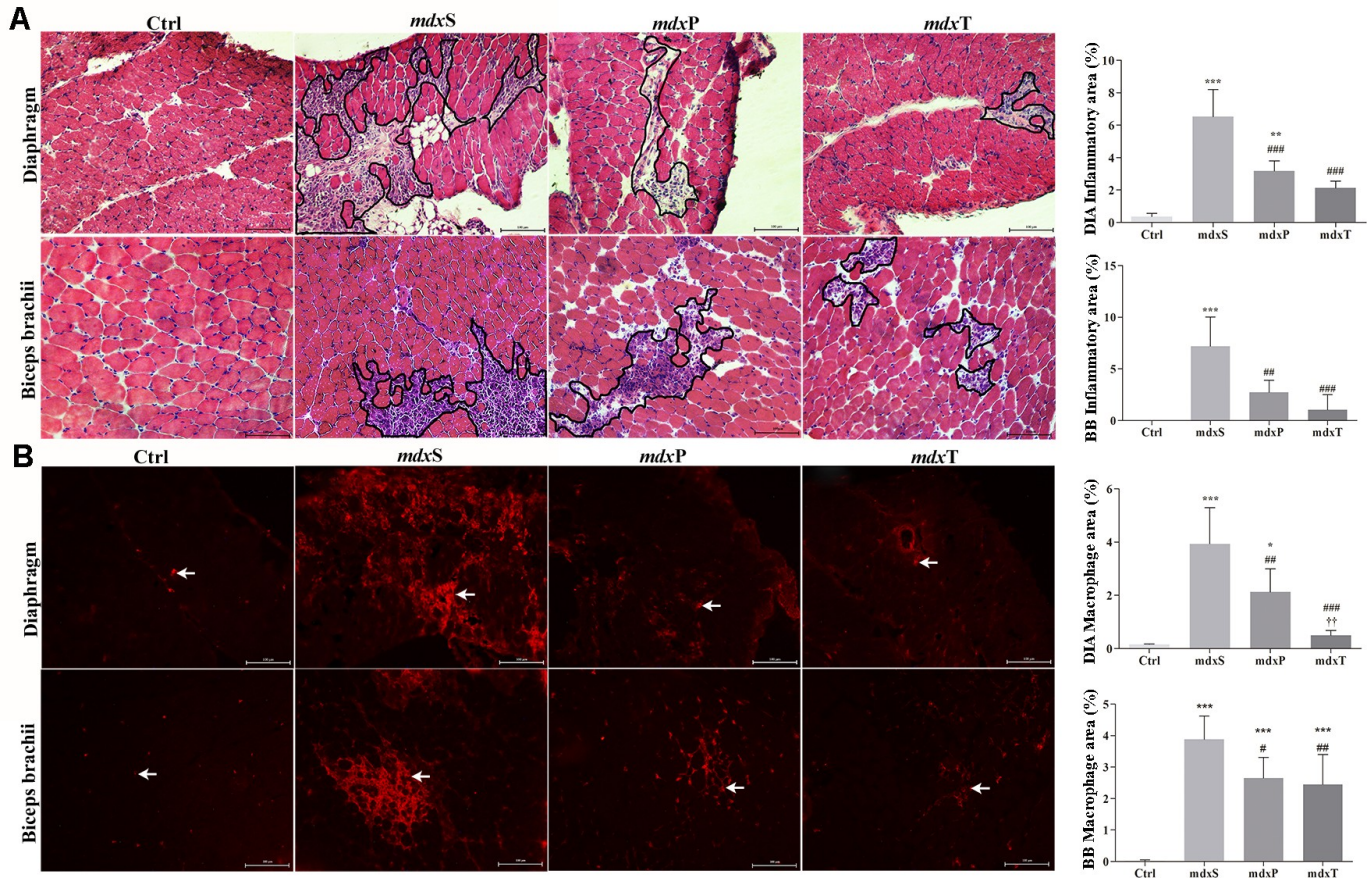
Inflammatory response morphology in dystrophic diaphragm and biceps brachii muscles. In (A), the outline indicates the representative area of inflammation in *mdx* mice. The graphs show the inflammatory area (%) in diaphragm (DIA) and biceps brachii (BB) of C57BL/10 mice (Ctrl), saline-treated *mdx* mice (*mdx*S), prednisolone-treated *mdx* mice (*mdx*P) and Tempol-treated *mdx* mice (*mdx*T). In (B) Macrophage infiltration (arrows) was determined by F4/80 immunohistochemistry. The graphs show the macrophage infiltration area (%) in the DIA and BB muscles of Ctrl, *mdx*S, *mdx*P and *mdx*T. All values expressed as mean ± standard deviation (SD). *P ≤ 0.05 compared with Ctrl group, **P ≤ 0.001 compared with Ctrl group, ***P ≤ 0.0001 compared with Ctrl group, ^#^P ≤ 0.05 compared with *mdx*S group, ^##^P ≤ 0.001 compared with *mdx*S group, ^###^P ≤ 0.0001 compared with *mdx*S group, ††P ≤ 0.001 compared with *mdx*P group (one-way ANOVA with Tukey’s post-hoc test).

The delimited inflammatory area (Fig 4A) and the macrophage infiltration (Fig 4B) analyses showed an increase on inflammatory process in the *mdx*S group (Fig 4A). Prednisolone and Tempol treatments significantly decreased the inflammatory area (DIA: 51.2% and 67.3%, and BB: 62% and 85.6%, respcetively; Fig 4A) and macrophage infiltration (DIA: 46% and 87.4% and BB: 25.1% and 30.7%, respectively; Fig 4B) in the DIA and BB muscleS of *mdx* mice compared to the saline-treated *mdx* mice.

**Fig 5:**
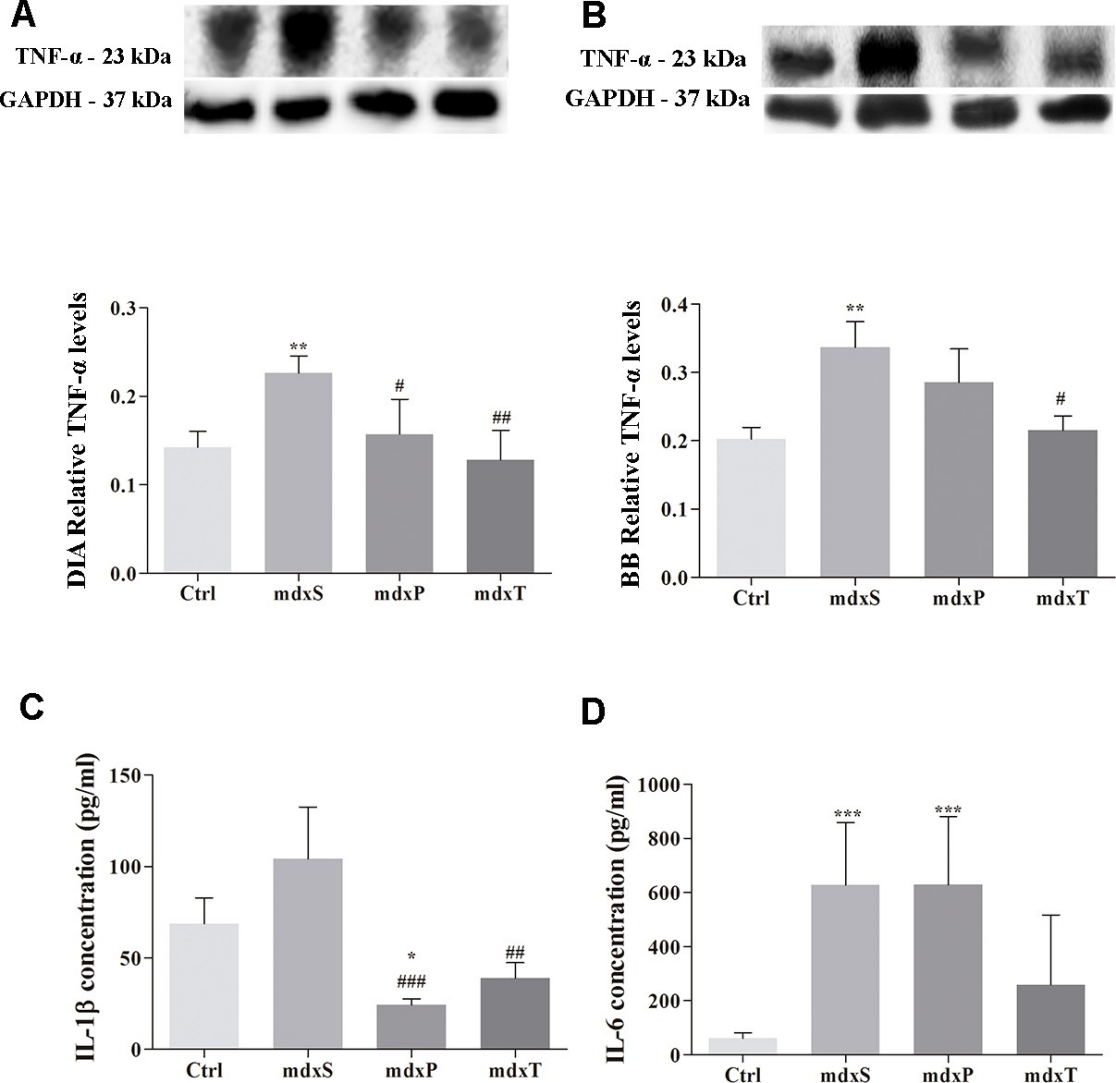
Factor and inflammatory cytokines involved in dystrophic process. In (A,B), western blotting analysis of tumor necrosis factor alpha (TNF-α). The blots of the proteins (top row) and of glyceraldehyde-3-phosphate dehydrogenase (loading control, bottom row), are shown. The graphs show protein levels in the crude extracts of diaphragm (DIA) and biceps brachii (BB) from C57BL/10 mice (Ctrl), saline-treated *mdx* mice (*mdx*S), prednisolone-treated *mdx* mice (*mdx*P) and Tempol-treated *mdx* mice (*mdx*T). The intensities of each band were quantified and normalized to those of the corresponding Ctrl. Relative values are expressed as mean ± standard deviation (SD). In (C) detection of IL-1β levels and in (D) detection of IL-6 levels in serum of Ctrl, *mdx*S, *mdx*P and *mdx*T. *P ≤ 0.05 compared with Ctrl group, **P ≤ 0.001 compared with Ctrl group, ***P ≤ 0.0001 compared with Ctrl group, ^#^P ≤ 0.05 compared with *mdx*S group, ^##^P ≤ 0.001 compared with *mdx*S group, ^###^P ≤ 0.0001 compared with *mdx*S group (one-way ANOVA with Tukey’s post-hoc test).

Regarding some molecular mechanisms involved in dystrophic inflammatory response, was observed an increase in TNF-α (DIA: 57.9%, BB: 61.4%), IL-1β (by 51.6%) and IL-6 (by 230%) levels in the saline-treated *mdx* mice compared to the control (C57BL/10) mice (Fig 5A, 5B, 5C and 5D). A marked reduction in these inflammatory mechanisms was observed in Tempol-treated *mdx* mice (Fig 5A, 5B, 5C and 5D).

### Tempol angiogenic effects

**Fig 6:**
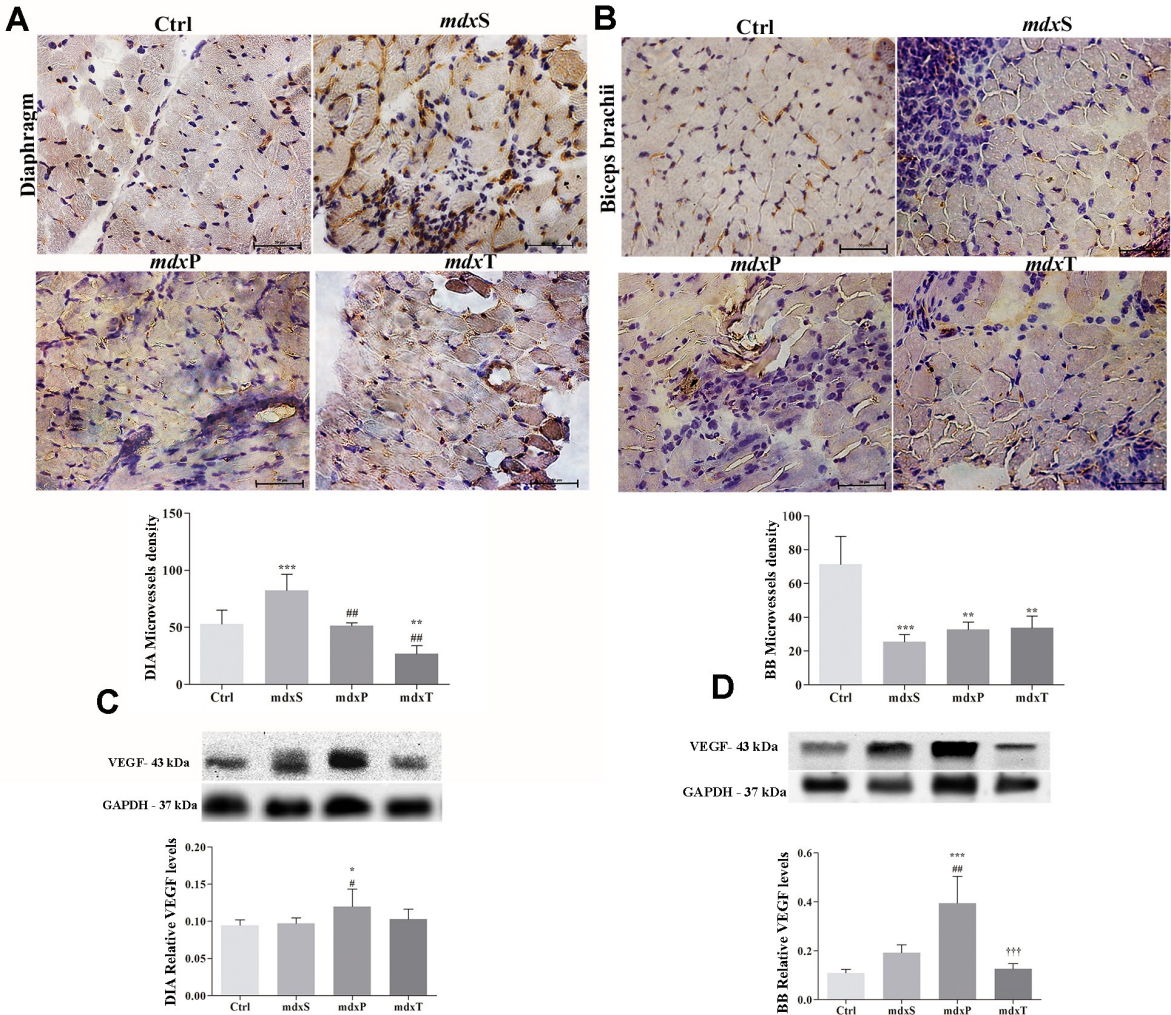
Angiogenesis in dystrophic diaphragm and biceps brachii muscles. In (A), diaphragm (DIA) and (B) biceps brachii (BB) cross-sections showing CD31 immunoreactivity (brown color) C57BL/10 mice (Ctrl), saline-treated *mdx* mice (*mdx*S), prednisolone-treated *mdx* mice (*mdx*P) and Tempol-treated *mdx* mice (*mdx*T). The graphs show determination of microvessel density by CD31 positive frequency in the DIA and BB of Ctrl, *mdx*S, *mdx*P and *mdx*T. In (C,D), western blotting analysis of vascular endothelial growth factor (VEGF). The blots of the proteins (top row) and of glyceraldehyde-3-phosphate dehydrogenase (loading control, bottom row), are shown. The intensities of each band were quantified and normalized to those of the corresponding Ctrl. All values expressed as mean ± standard deviation (SD). *P ≤ 0.05 compared with Ctrl group, **P ≤ 0.001 compared with Ctrl group, ***P ≤ 0.0001 compared with Ctrl group, ^#^P ≤ 0.05 compared with *mdx*S group, ^##^P ≤ 0.001 compared with *mdx*S group, †††P ≤ 0.0001 compared with *mdx*P group (one-way ANOVA with Tukey’s post-hoc test).

The Tempol angiogenic effect was evaluated in DIA and BB muscles of experimental groups by the positive microvessels for CD31 and VEGF levels (Fig 6A, 6B, 6C and 6D).

The positive microvessels by CD31 was significantly greater (by 36.8%) in DIA muscle from *mdx*S group compared to the control (C57BL/10) group and the Tempol and Prednisolone treatments significantly decreased this increase (Fig 6A). On the other hand, in the BB muscle, untreated and treated *mdx* groups showed a significant reduction in positive microvessels by CD31 compared to the control (C57BL/10) group (Fig 6B).

Regarding VEGF levels, was observed an increase on their levels in the DIA and BB muscles from *mdx*P group compared to Ctrl and *mdx*S groups (Fig 6A, 6B, 6C and 6D). Tempol treatment significantly decreased the VEGF levels in the dystrophic BB muscle compared to the *mdx*P group (Fig 6D).

## Discussion

Tempol is a redox-cycling (catalytic), metal-independent, and membrane permeable superoxide dismutase mimetic [15]. Beneficial Tempol effects have been observed in several diseases [15]. The advantageous Tempol effects in the dystrophic skeletal muscle of *mdx* mice were demonstrated in the results herein by means of gain muscle strength, muscle degeneration/regeneration reduction and inflammatory response decrease.

Liver morphology and function evaluated by ALT and AST levels showed that Tempol treatment does not present any toxic effect during the period and with the dose used in our experiments. Although, ALT and AST levels are commonly tested to screen for liver disease, some studies reported that these enzymes are increased in muscular dystrophy [16–18]. The increase of these aminotransferases levels in dystrophinopathy is more related to their release from damaged myofibers than to liver injury [16, 18]. So, the beneficial Tempol effect on ALT and AST levels also corroborates with our myonecrosis findings.

We observed that Tempol treatment induced an improvement in terms of myonecrosis, with increased muscle strength gain and a good correlation with the histological findings, consisting of a significant decrease in sarcolemmal integrity loss. The EUK-134, which has a Tempol-like function of acting as a catalytic mimetic of superoxide dismutase, also reduced the number of fibers with central nuclei and a partial rescue of force in the *mdx* diaphragm [19], thus strengthening our morphological and functional findings about indicators of dystrophic muscle damage. Already in agreement with our functional results, Burns and collaborators (2017) showed that chronic Tempol supplementation increases dystrophic diaphragm force generation, restoring the twitch and titanic contractions to values similar to wild-type values [7]. Still collaborating with our Tempol functional effect finding, it was also reported that the Tempol administration prevented tetanic force reduction, caused by the increase in superoxide muscle production, in rat muscle fibers [20].

We could also verify another important effect of Tempol treatment, which was its anti-inflammatory action. Both morphological and molecular inflammatory indicators, analyzed is this work, were reduced in the dystrophic animals after Tempol treatment. These results are congruent with several studies that have shown the anti-inflammatory effects of Tempol (e.g. Tempol blocked the release of cytokines from cultured human umbilical vein endothelial cells challenged with macrophage chemotactic protein-1 or interleukin-6 [21]; reduced the pleural exudation and the polymorphonuclear cell migration in a rat carrageenan-induced model of pleurisy [22] and reduced the inflammation in a rodent model of periodontitis [23]. Recently, Santos, Ribeiro and collaborators (2018) also reported that Tempol inhibited neutrophil kinase activities in phagocytic cells [24]. According to Gomez-Pinilla and collaborators (2010), the reduction in the expression of inflammatory markers observed after Tempol treatment makes this antioxidant a good candidate to be tested as a non-steroidal anti-inflammatory drug [25].

We also determined the Tempol effects of angiogenesis in dystrophic muscles by evaluating the positive microvessels for CD31 and VEGF levels. In these parameters, no beneficial action of Tempol was observed. Tempol's performance in angiogenesis and vascular perfusion is still not fully understood. There are studies demonstrating that Tempol stimulates angiogenesis, while others demonstrate an inhibitory effect [26, 27]. We also observed that the analyzed muscles, diaphragm and biceps brachii, had different behaviors regarding to CD31. The majority of studies showed the reduced vascular densities and impaired angiogenesis in limb muscles of young mdx mice [4, 15]. It is well documented that the progression of dystropathology is different in the *mdx* diaphragm with significant fibrosis by 9 months [8]. So, it is possible that the diaphragm in young mdx mice showed increased vascular density, a fact that might even justify the presence of late fibrosis. However, future studies are needed to confirm this hypothesis and clarify the interaction mechanisms of Tempol with the vascular pattern in the dystrophic muscle tissue.

In agreement with the Prednisolone effects observed in our study, previous work also demonstrated its beneficial actions by improvement of muscle function in DMD patients [28]. However, in *mdx* mice, the Prednisolone functional improvement occurs only in the initial periods of treatment and after prolonged use of glucorticoids, deterioration of muscular and cardiac function is observed [29].

Therefore, our interpretation is that the improvement in *mdx* muscle morphology and function promoted by Tempol, observed in our results and by Burns and collaborators (2017) [7], might be explained that apart from its antioxidant effects, it may also have anti-inflammatory actions, further supporting its use as a potential therapeutic strategy in DMD. Systemic administration of Tempol in humans has not been evaluated, however topical use has already been clinically tested and found to be effective against radiation-induced alopecia [30].
